# Early Coverage of Upper Extremity Electrical Injury Wounds

**DOI:** 10.5812/traumamon.6971

**Published:** 2012-10-10

**Authors:** Shahram Nazerani, Mehran Sohrabi, Amir Shirali, Tina Nazerani

**Affiliations:** 1 Department of Surgery, Firouzgar Hospital, Tehran, IR Iran, Tehran, IR Iran

**Keywords:** Electric Injuries, Reconstructive Surgical Procedures, Free Tissue Flaps

## Abstract

**Background:**

An appropriate and well-timed surgery has great impact on a patient’s treatment and can prevent further damage to partially injured structures which if untreated will be lost leading to severe disability. In the present study we report our experience with early coverage of electrically injured upper extremity vital structures with encouraging results.

**Objectives:**

The aim of this study was to evaluate the results of early flap coverage (less than two weeks) after electrical injury in the induced wounds of upper extremity.

**Materials and Methods:**

The records of electrically injured patients referred during a 10- year period to Firuzgar Medical Center were evaluated. After one or two sessions of debridement, the wounds were covered by distant or pedicled flaps and the results were evaluated according to the number of surgeries, complications and return to work time.

**Results:**

Thirty patients were registered in this study, mean age at the time of injury was 26.43 (SD = 10.41) years; 40% of patients had right upper extremity injury, 23.3% had left and 36.7% had bilateral injury. 43.4% of patients had no complications, amputation rate was 23.3% and nerve injury was seen in 13.3% of patients. Mean days of return to work was 132.57 (SD = 64.99). In 11 patients distant flaps were used, 9 patients with graft only and 7 patients had a combination of graft and regional flaps.The dominant hand involvement in electrical injury is very high.

**Conclusions:**

We suggest that the routine treatment protocols of serial debridement until all the wound acquires a bed of granulation tissue should be revised, because the vital structures such as tendons and nerves will have undergone dessication necrosis and a young worker will be crippled for life. Early coverage of partially injured vital structures is gaining acceptance and this paper confirms the above mentioned treatment protocol.

## 1. Background

An appropriate and well-timed surgery has great impact in a patient’s treatment strategy and can prevent further damage to partially injured structures which if untreated will lead to severe disability for the patient. Electrical injury leads to significant morbidity and mortality in the working age group. Most of electrical injuries are accidental and only a small percent of these injuries are suicidal attempts. Death due to electrical current occurs both with low and high voltage. Most deaths caused by electrical shock occur with low voltage home electricity (1-3). Almost 1000 deaths caused by electricity happen every year in USA. Electrical injury leads to 3000 admissions per year and comprises 3-4% of all burn admissions. Almost 40% of electrical injuries are fatal and lead to about 1000 deaths every year (4-6).

Electrical injury accounts for 5% of burn admissions in I.R. Iran, which is higher than the world statistics. Most of the electrical injuries are among construction workers who accidentally contact electrical wires during the demolition of old houses, a booming business in today's Iran; and the second group is ordinary people trying to gain unauthorized access to the cities’ electrical grid work. Electrical injury damages vary from superficial skin injuries to multi-organ involvement including heart, kidney, nervous system, eye and musculoskeletal system (7, 8).

One of most important body part that can be damaged due to electrical injury is the upper extremity which is the most common. This injury can have serious effects on the working age group and early reconstruction with local and distant flaps to protect vital structures is of utmost importance. After the resuscitation period, our protocol consists of debridement of obvious necrotic skin and muscles, without removing the exposed tendons, especially in the wrist area, protecting the tendons and vital structures by moist gauze dressing and after 4 to 5 days until the injury is stable enough to cover the defect with regional or distant flaps. In the wrist area we judiciously use soft tissue distraction or ligamentotaxis by pentagonal frame to maintain the tendon length and prevent future hand and wrist contractures. The exposed non-vital structures such as skin and muscle are covered by skin graft.

## 2. Objectives

The aim of this study was to evaluate the results of early flap coverage (less than two weeks) of electrical injury induced wounds of upper extremities.

## 3. Methods and Materials

This retrospective study included patients with electrical injury of upper extremities who were referred to Firuzgar hospital of Tehran from year 2001 until 2011. There were 30 patients and all of them were male. The relevant data such as site of electrical injury, entry and exit points, type of coverage, number of surgeries, complications, return time to work, amputation rate and death were entered in the database. The data were analyzed by SPSS v.18 software recording the average, prevalence percent, and standard deviation.

## 4. Results

Overall, 30 patients with upper limb electrical injuries were included in the study. All were male and from 2 to 46 years-old with average age of (SD = 10.41) 26.43 years. Involved limb in 12 patients (40%) was the right side, in 7 patients (23.3%) was the left and in 11 patients (36.7%) was bilateral. Involved parts were “hand” in 26 patients, “arm and forearm” in 6 patients and bilateral extremity involvement in 2 patients. The exit point in all the patients except one case of facial exit wound was the lower limb. Table 1 shows the relevant data. The numbers of reconstructive procedures after the early debridement were as follows:

**Table 1:** Patients’ data

Age, y	Surgeries	Involved Extremity	Return to Work, d	Complication	Method of Surgery
**46**	1	Right Upper Extremity Tenar atrophy	120	Median nerve injury	PL to EPL transfer
**18**	2	Right hand	****	Mid arm amputation	Amputation and revision
**23**	2	Upper Extremities	30	Low ROM	Reverse Flap and Graft of both fore arms
**31**	3	Right forearm and hand left hand	28	-	Abdominal Flap Skin Graft
**22**	2	Right hand	285	Ulnar nerve injury and ulnar artery bleeding	Free abdominal Flap
**36**	4	Both hands	****	Amputation	left mid forearm amputation STSG
**34**	1	Right median nerve and FDS,FDPand index injury	70	-	Radial venous Flap
**44**	1	Right Extremities	180	-	STSG
**23**	1	Left index	14	-	advance Flap
**32**	2	Left first to third fingers	180	Thumb amputation	Amputation of thumb osteocutaneous Flap distal Flap transfer to long finger and index FTSG
**2**	4	Flexion contracture of left index. Distraction of DIP andPIP	137	Nail injury	Bone Graft and abdominal Flap
**26**	7	Right Upper Extremity and left forearm	97	Right arm amputation	Dorsal Skin Flap mesh Skin Graft for left forearm
**27**	1	Left thumb and index, contracture in first web	125	-	Skin and digital nerve Graft from medial arm
**10**	2	Both hands	180	-	STSG
**27**	5	Right Upper Extremity	250	reduceROM	Abdominal Flap Groin Flap for fingers
**31**	1	Right hand	78	Median nerve injury	Abdominal Flap
**21**	3	Right Upper Extremity	145	Nerve injury	Abdominal Flap STSG
**30**	1	Right thumb	162	-	STSG
**22**	2	Left hand	55	Nail injury	distal Flap transfer to long finger
**33**	1	Right Upper Extremity	192	-	Abdominal Flap Skin and index nerve Graft
**19**	3	Right hand and forearm	181	Thumb and index amputation	STSG
**46**	1	Left hand	69	Reduce finger ROM in extension	Abdominal Flap
**6**	2	Left thumb and index	176	-	STSG
**14**	1	Left forearm	111	Reduce ROM	Abdominal Flap
**38**	3	Left thumb and right forearm	98	Thumb amputation	Amputation
**26**	1	Both hands	163	-	Abdominal Flap
**27**	5	Right forearm and left hand	128	Left thumb amputation	Abdominal Flap
**26**	2	Both hands	199	-	STSG
**27**	3	Right arm and forearm, left thumb	145	-	Abdominal Flap – STSG
**26**	4	Right thumb and left hand	114	-	Abdominal Flap

**Figure 1:**
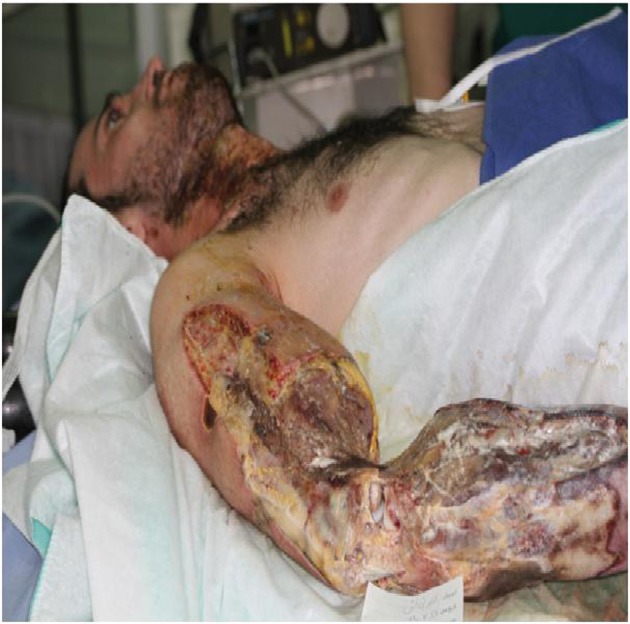
A Patient with Electrical Burn Leading to Amputation

One surgery in 11 patients (36.7%), two surgeries in 8 patients (26.7), three surgeries in 5 patients (16.7%) four surgeries in 3 patients (10%), five surgeries in 2 patients (6.7%), seven surgeries in 1 patient (3.3%). Return to work time varied from 14 to 285 days with an average of (SD = 64.99) 132.57 days. Complications observed were: none in 13 patients (43.3%), residual nerve injuries in 4 patients (13.3%), amputation in 7 patients (23.3%), total hand function reduction in 5 patients (16.6%) and nail defect in 1 patient. Figure 1 shows one of patients with electrical burn that led to amputation.

**Figure 2:**
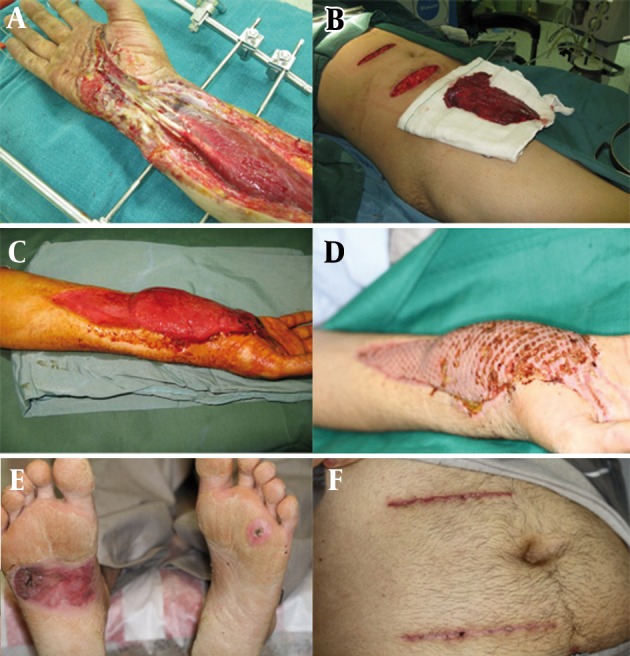
A Construction Worker with Electrical Burn During Work. A) Tendons Were Exposed and Frame Were Used to Fix the Hand. B) Rectus Muscle with Arteries is Ready to Transfer. C) After 6 Days Muscle is Ready to Graft D) Graft is Done and the Skin is Clean. E) Exit Site F) Donor Site

Distant and free flaps were used in 11 patients (36.7%), in 9 patients (30%) skin graft and in 7 patients (23.3%) a combination of skin graft and flaps was used. Time to return to work in flaps was 106.45 (SD = 71.06), in the combination of flap and graft was154.14 (SD = 67.28) and in the other cases (SD = 6.36) 115.50 days. The flaps that were mostly used included 45% of abdominal flaps, followed by 30% reverse forearm flap, 20% free muscle flap, and 5% musculocutaneus flap. One case presented in Figure 2 who was a construction worker with electrical burn during work which was referred after 4 days. He had a clean wound however tendons and nerves had dessication necrosis.

## 5. Discussion

Electrical injury is one of the most debilitating injuries of the modern Societies’ Traumas. The involvement of dominant upper extremity in the working age group of patients makes it more worrisome. The routine protocol of serial debridement of necrotic tissues until the entire wound has granulated has unsatisfactory outcomes and several groups are advocating early coverage of electrical injury wounds (9). This protocol has produced better results than the old method and our study confirms the superiority of this treatment protocol. The primary goal of reconstruction of the upper extremity is to restore function. Burns to the upper extremity lead to deformities and limit function, thus affecting all activities of daily living. Numerous methods have been described to overcome the dynamic (early phase) and static (late phase) complex scar contracture (10). Scars cause tendon adhesions, contractures, deep adipose tissue stiffness, and limitation of major joint range of motion (11). We have used soft tissue distraction with pentagonal frame to overcome the dynamic phase in these patients.

A variety of flaps are at the disposal of hand surgeon and abdominal flap followed by reverse radial forearm flap have been our preferred coverage modalities. Free Flaps offer the advantages of early mobilization of the upper extremity, excellent functional results, and less concern about limited donor skin availability (11). Abdominal flap was the flap of choice for most of our patients who had had either a forearm fasciotomy or forearm injury preventing the use of forearm flap for wrist area coverage.

Early coverage of electrically injured upper extremity vital structures can have superior results to conventional method of serial debridement and late coverage due to the loss of partially damaged vital structures. Coverage of exposed tendons and nerves even when grossly seem to have no vascularity can preserve these structures with a 100% success in free flaps in this series; and last but not least, the “old protocol” of vascular injury beyond the zone of injury and fear of vascularized local flaps needs to be revised.
